# Pictorial essay: MRI evaluation of endometriosis-associated neoplasms

**DOI:** 10.1186/s13244-023-01485-8

**Published:** 2023-09-07

**Authors:** Louise Radzynski, Louis Boyer, Myriam Kossai, Anne Mouraire, Pierre-François Montoriol

**Affiliations:** 1 Department of Radiology, University Hospital of Clermont-Ferrand, Clermont-Ferrand, France; 2 Department of Pathology, Cancer Center of Clermont-Ferrand, Clermont-Ferrand, France; 3 Department of Radiology, Cancer Center of Clermont-Ferrand, Clermont-Ferrand, France

**Keywords:** Endometriosis, Neoplasm, MRI, Endometrioid carcinoma, Clear cell carcinoma

## Abstract

Endometriosis is a frequent pathology mostly affecting women of young age. When typical aspects are present, the diagnosis can easily be made at imaging, especially at MRI. Transformation of benign endometriosis to endometriosis-associated neoplasms is rare. The physiopathology is complex and remains controversial. Endometrioid carcinoma and clear cell carcinoma are the main histological subtypes. Our goal was to review the main imaging characteristics that should point to an ovarian or extra-ovarian endometriosis-related tumor, especially at MRI, as it may be relevant prior to surgical management.

**Key points**

• Transformation of benign endometriosis to endometriosis-associated neoplasms is rare.

• MRI is useful when displaying endometriosis lesions associated to an ovarian tumor.

• Subtraction imaging should be used in the evaluation of complex endometriomas.

**Supplementary Information:**

The online version contains supplementary material available at 10.1186/s13244-023-01485-8.

## Introduction

Endometriosis is a condition characterized by the growth of functional ectopic endometrial glands and stroma outside the uterus. It is a frequent and still underestimated disease, affecting about one in ten women in their reproductive years [1] but also older women following menopause [2].

Malignant transformation of endometriosis is deemed extremely rare, with an estimated prevalence of less than 1% [3].

As a tertiary center specialized in gynecological oncology, we encounter cases of endometriosis-associated malignancy on a regular basis. The aim of this pictorial essay is to provide the key features which enable radiologists to raise a suspicion of endometriosis-associated neoplasms at magnetic resonance imaging (MRI).

## Endometriosis and associated neoplasms

Endometriosis is a benign disease. Nevertheless, this condition shares some characteristics with malignant neoplasms, such as development of local and distant foci and attachment to or invasion of other tissues, with subsequent damage to the target organs. Furthermore, endometriosis exhibits recurrence, unregulated cell proliferation, and estrogen-dependent growth [4]. Endometriotic epithelial cells have a higher turnover than their stromal counterpart due to cyclic bleeding and have much higher mutation frequencies. Recent studies have shown that endometriosis lesions carry mutations similar to those of cancer cells [5]. Endometriosis-associated neoplasms encounter molecular abnormalities including the activation of the oncogenic KRAS and PI3K pathways and inactivation of tumor suppressor genes [6]. Oxidative stress, chronic inflammation, and hyper endo/exogenous estrogenism also play a key role in carcinogenesis [5, 6].

Concerning the particularities of endometriosis-related cancers, the patients are often younger [6–8], diagnosed at an early FIGO stage, the neoplasm is most likely unilateral [8], and the prognosis is better than cancers without associated endometriosis [9, 10].

The two prevalent histological subtypes are endometrioid adenocarcinoma (67% of cases) and clear cell carcinoma (15% of cases) [11]. Other histological subtypes are less common and include serous adenocarcinoma, mucinous tumors, borderline tumors, and sarcoma [12, 13].

The definitive histological diagnosis is established face to concomitant lesions (Additional file 1: Figs. S1 and S2) of benign endometriosis and malignant components [11]. However, this association is not always found.

Apart from malignant transformations of endometriosis lesions, patients with endometriosis are overall more at risk of developing ovarian or extra-ovarian neoplasms, especially endometrial or breast cancer [14].

## Imaging

### Ultrasound (US)

Ultrasound is the first-line imaging modality for the detection of endometriosis. An endometrioma is easy to recognize, corresponding to a cyst with low level of internals echoes [15]. US is also effective in assessing the depth of digestive involvement (Additional file 1: Fig. S3). However, it is an operator-dependent modality which requires experienced hands. In case of increasing in size, the presence of irregular solid component, and blood flow in Doppler mode, the operator should suspect a malignant ovarian lesion at US [16, 17] and require further investigations using cross-sectional imaging [18].

### Computed tomography (CT)

CT scans are often performed for another indication but may nevertheless identify suspicious ovarian lesions and guide the diagnosis [19]. CT scans can show a papillary projection in a cystic mass or enhancing solid mural nodules [6]. Its main interest consists of evaluating the extension of the disease, especially by detecting lymph node involvement, peritoneal extension, or lung metastases.

### Magnetic resonance imaging (MRI)

MRI is an effective but less accessible modality. Not only does it allow the diagnosis of benign ovarian cyst and deep pelvic endometriosis and associated malignancy, but also the appreciation of loco-regional extension of the disease. It is also useful in detecting recurrences after medical or surgical treatment.

The recommended protocol to explore the pelvic endometriosis has been updated in 2017 by the European Society of Urogenital Radiology [20] and includes T2-weighted images in all orthogonal three planes and T1-weighted sequences with and without fat suppression.

Typical ovarian endometrioma displays moderate low signal intensity on T2-weighted images, called the “T2 shading effect” due to repeated intracystic bleeding. The specificity of this sign is > 90% [9]. Homogeneous, high signal intensity is shown on fat-saturated T1-weighted sequences [1].

Extra-ovarian, deep-pelvic endometriosis usually presents as small hemorrhagic foci or retractile fibrous thickening, especially affecting the torus uterinum and uterosacral ligaments (Additional file 1: Fig. S3).

Intravenous injection of gadolinium chelates is not mandatory in the setting of typical endometriosis, but it should be performed in the presence of a solid component within an endometrioma, in order to better characterize the lesion [18, 21]. Thus, for the exploration of a complex ovarian mass, it is recommended to add the following sequences [16, 22]:

- Diffusion-weighted imaging (DWI) with at least a high *b* value of 800, in which solid, cellular portions display high signal intensity with associated low signal on the apparent diffusion coefficient (ADC) map.
- Dynamic contrast-enhanced (DCE) imaging: in cases of malignant lesions, solid components typically enhance according to a type 3-dynamic curve, i.e., higher than that of the normal myometrium.

These features help classify the lesion according to the recently validated Ovarian-Adnexal Reporting Data System Score at MRI (O-RADS MRI), ranging from 1 to 5. An O-RADS 5 lesion with solid nodules, diffusion restriction, and enhancing pattern following a type 3 dynamic curve presents a high risk of malignancy > 90% [21].

Contrast-enhanced dynamic subtraction images are obtained by using a gradient-echo sequence [22] and subtractions between enhanced and unenhanced fat-saturated T1-weighted images. They are helpful for distinguishing the true enhancement of solid nodules that may be hard to depict within the hemorrhagic cystic fluid of endometriomas on T1-weighted sequences [5].

The typical presentation of a unilocular ovarian mass with hypointensity of the cystic components on T2-weighted imaging may help to distinguish endometriosis-associated malignancy from non-endometriosis-associated malignancy [8].

In patients with a history of endometriosis, increasing in size endometrioma or loss of shading on T2-weighted images [6, 11] should raise concern over a potential malignant transformation. The loss of shading on T2 images is thought to be due to fluid secretions by the malignant components, diluting the chronic hemorrhagic content.

Table 1 summarizes the key MRI features of typical endometrioma and suspicion of endometriosis-associated neoplasm.

**Table 1:**
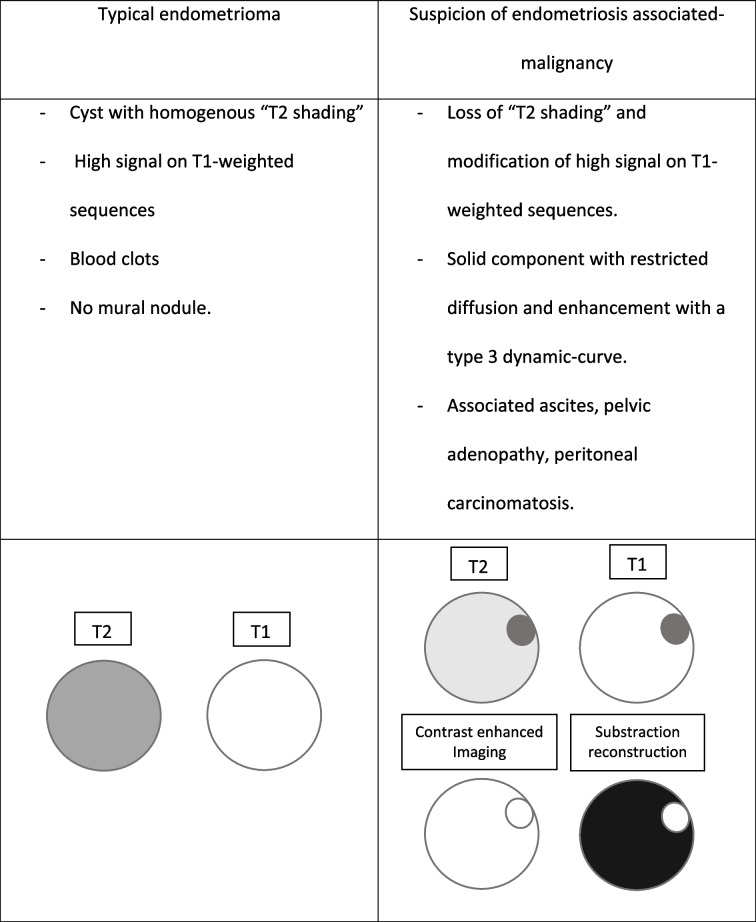
Schematic MRI features of typical endometrioma and suspicion of endometriosis-associated neoplasm

**Fig. 1:**
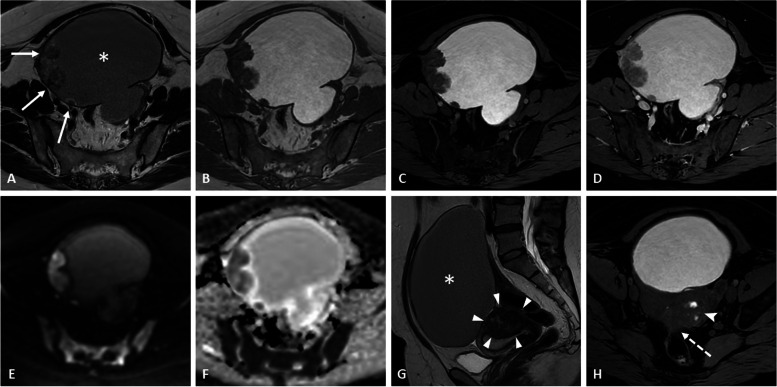
Endometrioid adenocarcinoma within a right ovarian endometrioma in a 43-year-old woman without any medical history. Axial T2-weighted image (**a**) shows a large ovarian cystic mass with chronic bleeding (asterisk) displaying a “T2-shading” appearance and peripheral wall nodules (arrows). The axial T1-weighted (**b**) and fat-suppressed T1-weighted (**c**) images confirm the hemorrhagic cystic content. Enhancement of the nodules is seen on fat-suppressed T1-weighted following gadolinium chelates injection (**d**). The axial diffusion image (**e**) shows the high signal intensity regarding neoplastic nodules, with corresponding low intensity on the ADC map (**f**). Sagittal T2-weighted image (**g**) shows features of pelvic endometriosis with uterine adenomyoma (arrowheads) and several hemorrhagic foci (arrowhead) with anterior rectal wall involvement (dotted arrow) on axial fat-suppressed T1-weighted image (**h**)

**Fig. 2:**
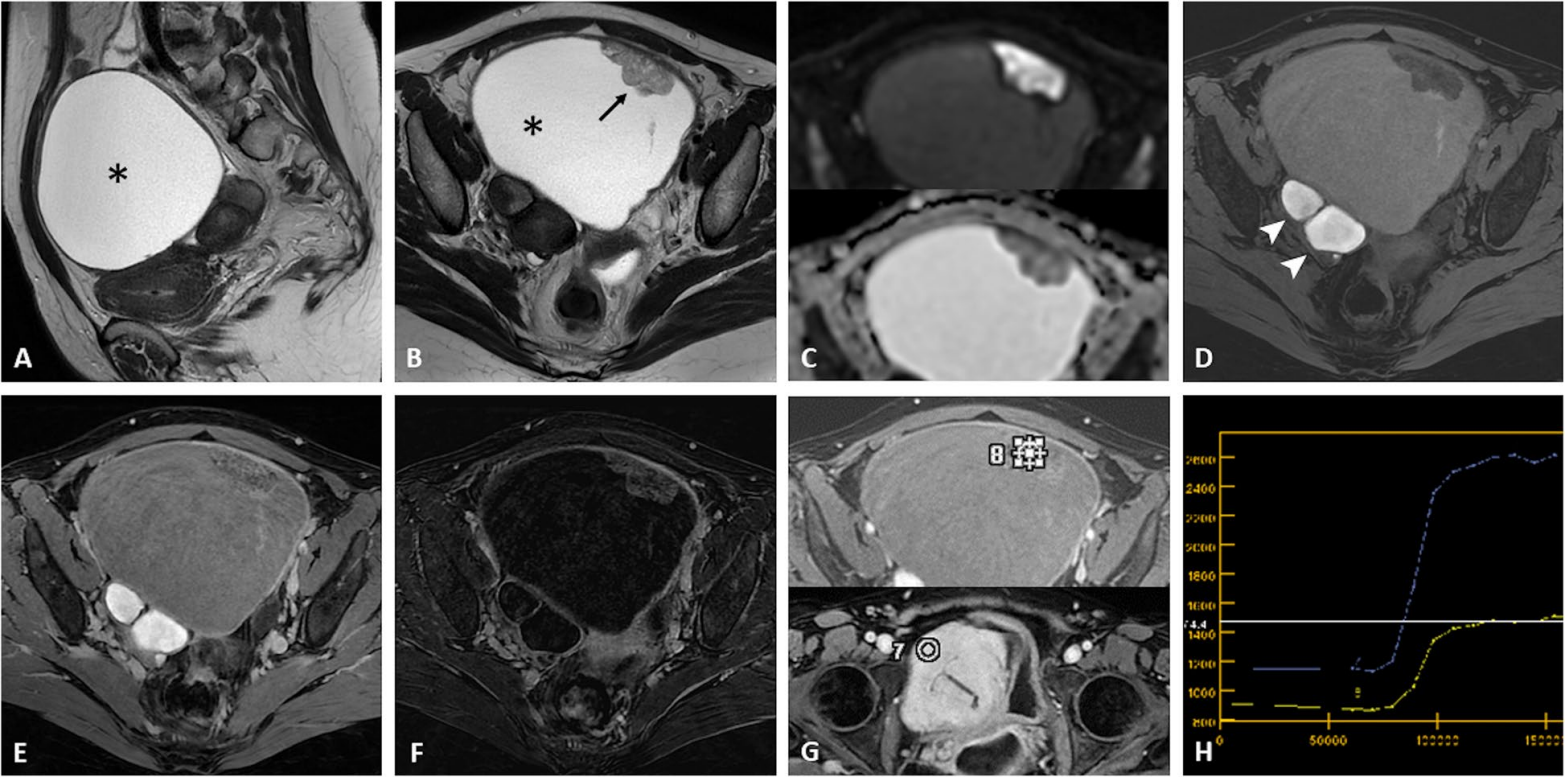
Ovarian endometrioid adenocarcinoma within an endometrioma in a 39-year-old woman with a known history of endometriosis. Sagittal T2-weighted image (**a**) shows a left ovarian cystic mass (asterisk) without a T2-shading effect. Axial T2-weighted image (**b**) shows a mural nodule (arrow) with restricted diffusion on a high *b800* value image and corresponding ADC map (**c**). The axial fat-suppressed T1-weighted image (**d**) displays a typical right ovarian endometrioma (arrowheads). Axial contrast-enhanced fat-suppressed T1-weighted image (**e**) shows a light enhancement of the nodule, better displayed on the subtraction sequence (**f**). Dynamic contrast enhancement analysis of the nodular portion (**g**, **h**) demonstrates a type 3 curve (blue curve, located above normal myometrium in the yellow curve), consistent with a malignant etiology

**Fig. 3:**
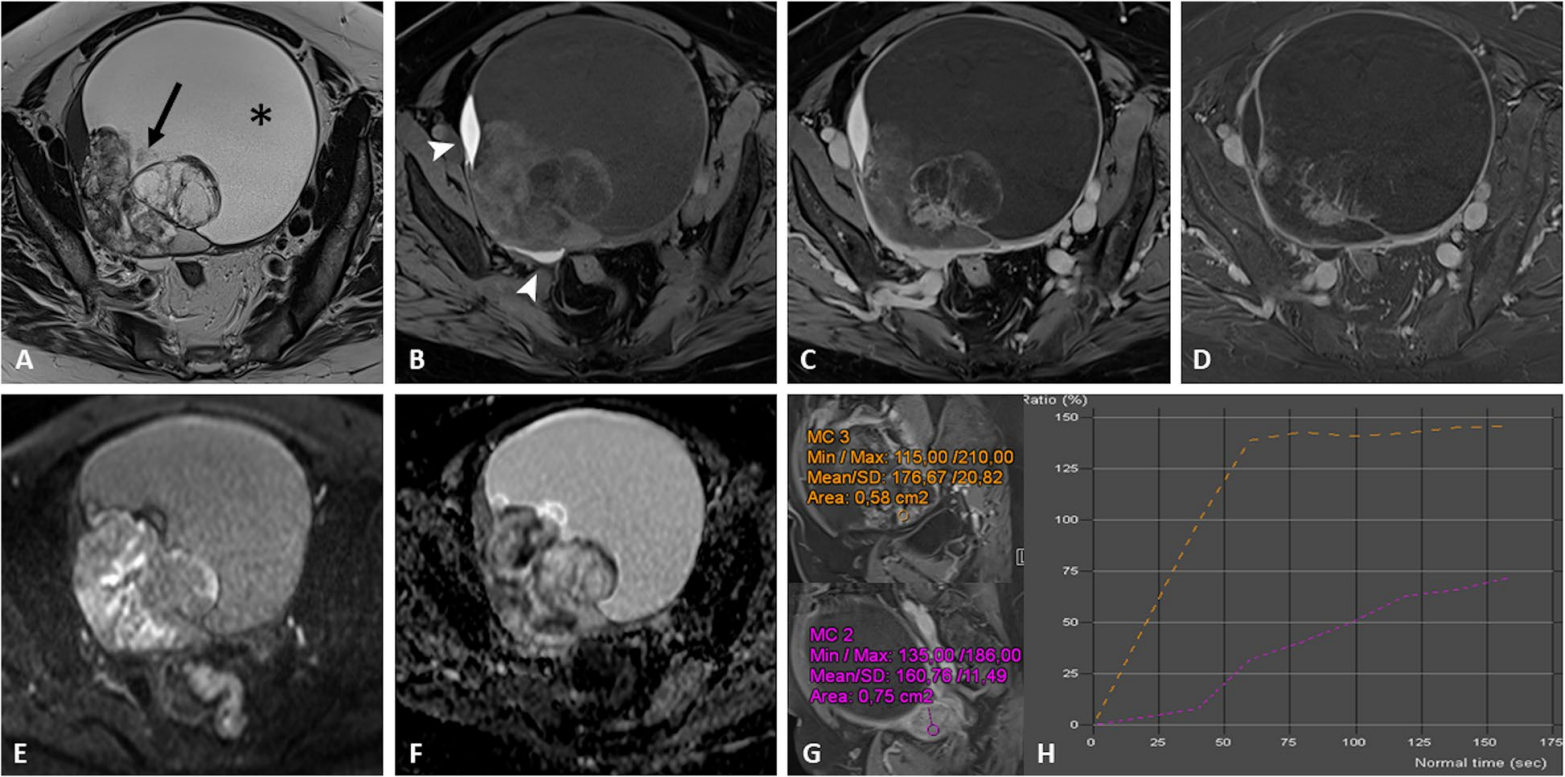
Clear cell carcinoma on right ovarian endometrioma in a 66-year-old woman with a palpable abdominal mass. Axial T2-weighted image (**a**) shows a mixed cystic (asterisk) of the right ovary containing a large peripheral solid portion (arrow). Hemorrhagic component (arrowhead) corresponding to endometriomas are displayed on axial fat-suppressed T1-weighted image (**b**). Enhancement of the solid portions is seen on axial contrast-enhanced fat-suppressed T1-weight image (**c**) and corresponding subtraction image (**d**). Diffusion-weighted imaging (**e**, **f**) shows restricted diffusion of neoplastic portions. Dynamic contrast enhancement analysis (**g** high *b* value and **h** ADC map) shows a type 3 curve regarding the solid portions (orange curve) located above the myometrial reference curve (pink curve), consistent with malignancy

**Fig. 4:**
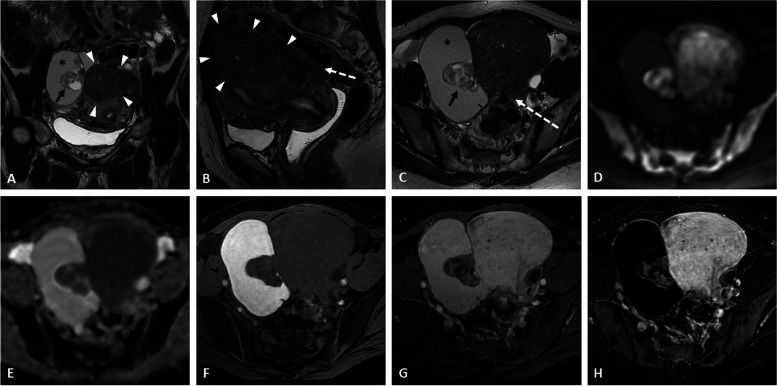
High-grade serous adenocarcinoma within an endometrioma in a 48-year-old woman with a known history of endometriosis. Coronal (**a**), sagittal (**b**), and axial (**c**) T2-weighted images show severe uterine adenomyosis (white arrowheads) and deep endometriosis involving the posterior compartment (dotted arrows). There is a mixed cystic right ovarian mass (asterisk) with intermediate signal intensity on T2 images (shading effect) containing a mural nodule (black arrows). Diffusion-weighted imaging (**d** high *b* value and **e** ADC map) shows a restricted diffusion within the mural nodule. Axial fat-suppressed T1-weighted image (**f**) shows the typical features of ovarian endometrioma. Heterogeneous enhancement of the nodule is displayed on fat-suppressed contrast-enhanced T1 image (**g**) and subtraction sequence (**h**)

**Fig. 5:**
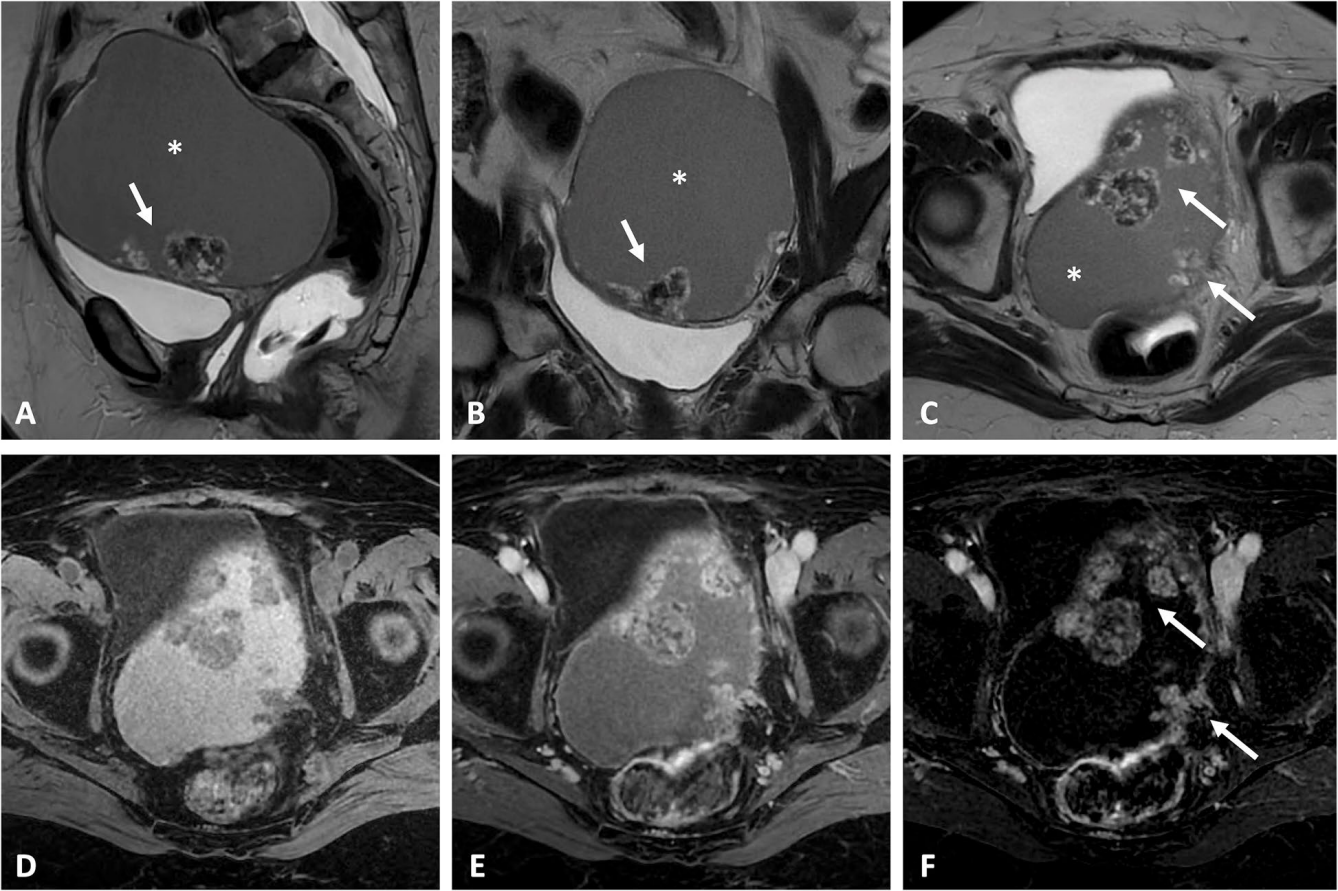
Borderline seromucinous tumor within an ovarian endometrioma in a 77-year-old woman with a long-standing history of endometriosis and previous hysterectomy for cervical cancer. Sagittal (**a**), coronal (**b**), and axial (**c**) T2-weighted images show a mixed ovarian mass filled with typical intermediate signal intensity content (*“shading” effect), containing multiple mural solid nodules (arrows). Axial fat-suppressed T1-weighted image (**d**) shows the homogeneous hemorrhagic fluid classically found within ovarian endometrioma. Heterogeneous enhancement of the nodules is displayed on fat-suppressed contrast-enhanced T1 image (**e**), better seen on subtraction sequence (**f**)

There is no true discriminating feature to differentiate endometrioid adenocarcinoma from clear cell carcinoma or other malignant pathology (Figs. 1, 2, 3, 4, and 5 and Additional file 1: Figs. S4 and S5). Nevertheless, according to some studies, clear cell carcinoma tends to have a larger tumor size and higher ADC values on DWI with infiltrative growth pattern, heterogeneous signal, and associated ascites [23], whereas endometrioid carcinomas have more multifocal or concentric mural nodules and a higher height-to-width ratio [24].

**Fig. 6:**
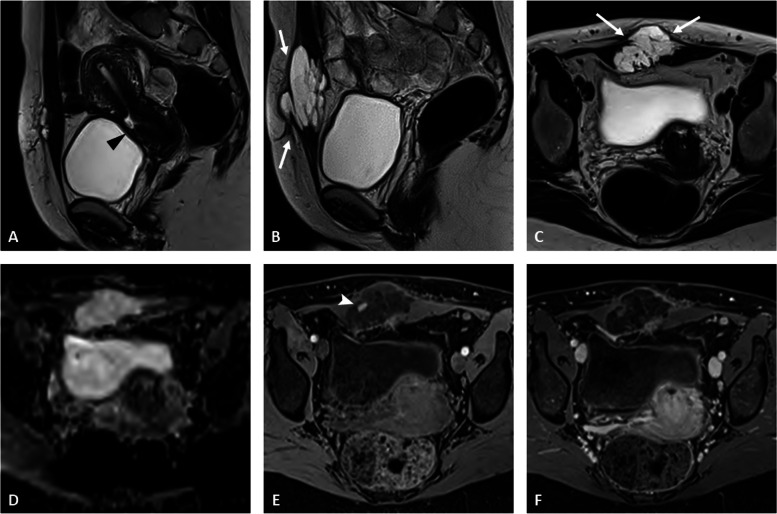
Clear cell adenocarcinoma of the abdominal wall in a 44-year-old woman with a history of cesarean section. Sagittal T2-weighted sequence (**a**) shows the scar on the anterior uterine isthmus (black arrowhead). Sagittal (**b**) and axial T2-weighted (**c**) images show a multiloculated cystic lesion developed within the anterior abdominal wall (arrows), infiltrating the right rectus abdominis muscle, without obvious diffusion restriction (**d**). The axial fat-suppressed T1-weighted image shows a small hemorrhagic spot (arrowhead) within the cyst (**e**). Diffuse enhancement of the cystic cavity wall and internal septae is seen on fat suppressed-enhanced T1 image (**f**). There was no surgery-associated pelvic endometriosis in this patient.

**Fig. 7:**
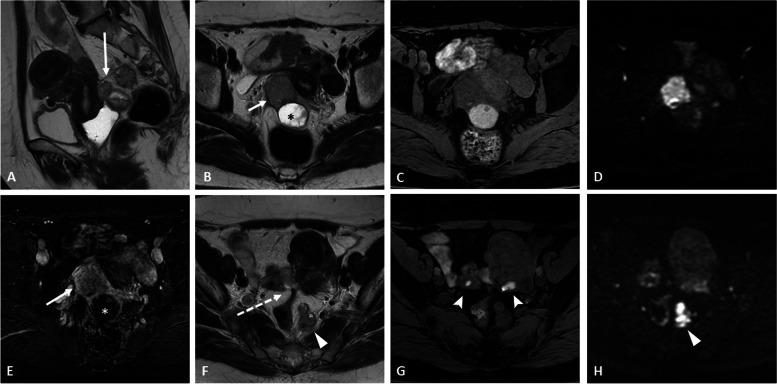
Endometrioid adenocarcinoma developed on deep pelvic endometriosis in a 58-year-old woman with a history of infertility. Sagittal (**a**) and axial (**b**) T2-weighted images show a large heterogeneous mass obliterating the Douglas cul-de-sac composed of solid (white arrows) and cystic (asterisk) portions. Axial fat-suppressed T1-weighted image shows hemorrhagic content within the cystic cavity (**c**). Diffusion-weighted imaging (**d** high *b* value) shows a high signal intensity within the solid portion with corresponding enhancement on axial enhanced fat-suppressed T1 image (**e**). Axial T2-weighted sequence at a higher level (**f**) shows the features of deep pelvic endometriosis of the posterior compartment (dotted arrow) and a metastatic nodule of the mesorectum (arrowhead). “Kissing ovaries” containing small-sized endometrioma (arrowheads) are seen on corresponding fat-suppressed T1-weighted image (arrowheads (**g**)). Restricted diffusion within the mesorectum metastasis is also seen (**h**)

Similar to endometriosis, associated cancers may be extra-ovarian (Figs. 6 and 7). The most commonly affected site is the abdominal wall in scarred areas, especially Cesarian scars [25]. In these patients, the abdominal wall involvement is usually isolated, rarely associated to pelvic endometriosis [26].

#### Pitfalls

**Fig. 8:**
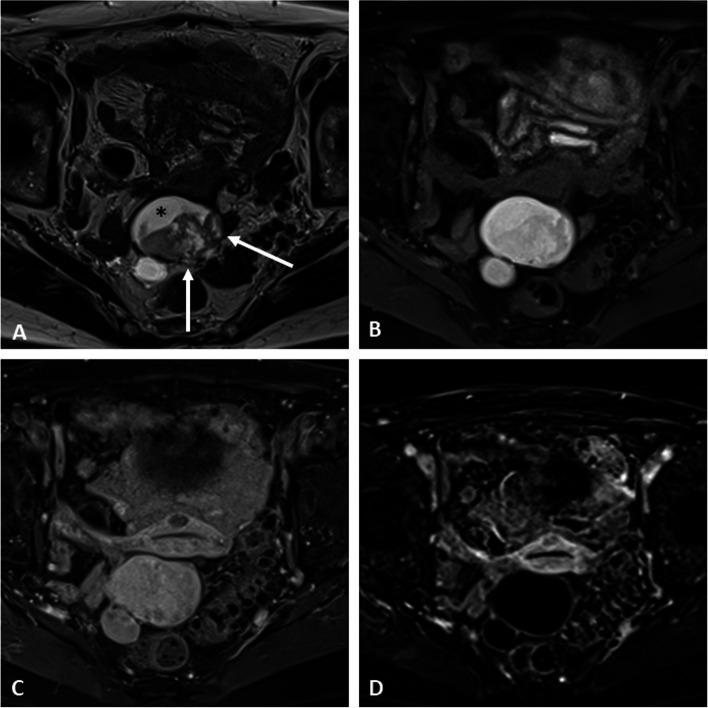
Differential diagnosis: right ovarian endometrioma in an 84-year-old woman with a history of breast cancer. Axial T2-weighted image (**a**) shows a right ovarian mixed lesion containing simple fluid (asterisk) and solid components (arrows). Due to the hemorrhagic filling, potential enhancement of the solid portion is difficult to appreciate between unenhanced (**b**) and enhanced (**c**) fat-suppressed T1 images, whereas the subtraction sequence perfectly displays the lack of enhancement of the solid component (**d**). The patient underwent surgery: pathology analysis confirmed the presence of blood clots within a benign endometrioma

**Fig. 9:**
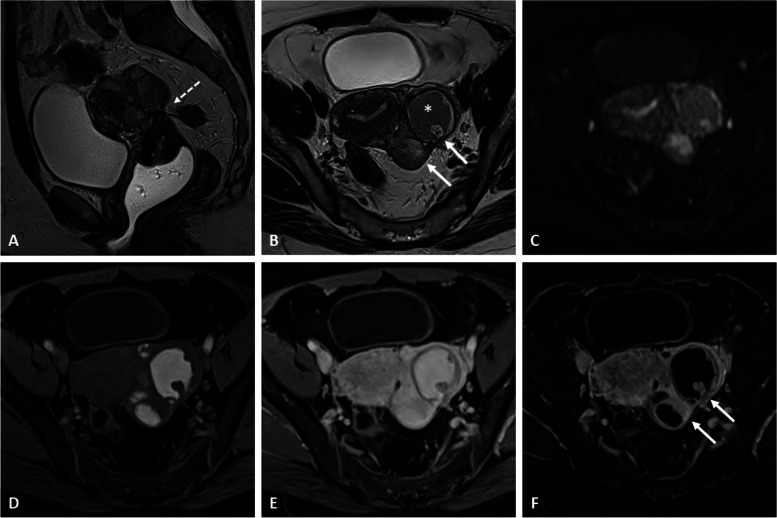
Differential diagnosis: atypical ovarian endometrioma without malignancy in a 50-year-old woman. Sagittal T2-weighted image (**a**) shows the thickening of the torus uterinum with adhesions to the anterior wall of the recto-sigmoid (dotted arrow). Axial T2-weighted image (**b**) shows a left ovarian cystic lesion (asterisk) with T2-shading effect and endocystic peripheral thickening and a polypoid nodule (arrows), both exhibiting diffusion restriction (**c**). Axial fat-suppressed T1-weighted image (**d** high *b* value) confirms the typical features of endometrioma. Axial contrast-enhanced fat-suppressed T1-weighted (**e**) and subtraction sequence (**f**) demonstrate significant enhancement of the solid portions (arrows). The patient underwent surgery, and the definitive histological result was a simple endometrioma with endocystic endometrium lining in the proliferative phase, without any associated malignancy

**Fig. 10:**
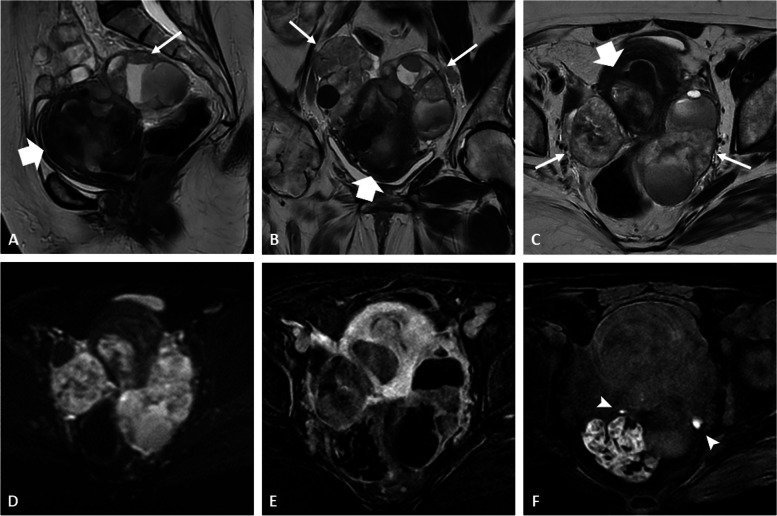
Differential diagnosis: endometrial carcinoma with bilateral ovarian metastases in a 57-year-old woman with endometriosis. T2-weighted images in the sagittal (**a**), coronal (**b**), and axial (**c**) plane show a large intra-uterine mass (thick arrows) and bilateral ovarian masses (thin arrows). All lesions display restricted diffusion (**d** high *b* value) and enhancement on axial contrast-enhanced fat-suppressed T1-weighted images (**e**). Small-sized hemorrhagic foci within the ovarian masses are shown on fat-suppressed T1 weighted image (arrowhead in **f**), corresponding to superficial endometriosis lesions on the ovarian surface. Surgery confirmed a high-grade endometrial carcinoma with ovarian metastases and underlying endometriosis

The main pitfalls at MRI are as follows:

- *Clot dark spot sign*: the presence of a well-defined, markedly hypointense foci within an ovarian cyst on T2-weighted images, corresponding to a blood clot. This sign has a high specificity and is useful for differentiating endometriomas from hemorrhagic cysts in which it is usually absent [27] (Fig. 8 and Additional file 1: Fig. S6).
- *Enhanced endocystic nodules* [28], including the following:
  ➔ Polypoid endometriosis (Fig. 9), which affects more commonly post-menopausal women with hormone replacement therapy. Similar to the uterine endometrium, it appears as a hyperintense T2-weighted polypoid nodule with hypointense peripheric rim, contrast enhancement pattern mirroring the enhancement of the uterine endometrium, and classically lack of diffusion restriction [29].
  ➔ Cystadenofibroma frequently appears as a multiseptated predominantly cystic mass with variable amounts of solid nodular components. The individual locules may contain hemorrhage or protein content which will modify the T1/T2 intensity cystic signal. The solid component typically displays marked T2 hypointensity, with no restriction in diffusion-weighted imaging (also called “dark-dark appearance”) and progressive enhancement with a type I perfusion curve [30].
  ➔ Decidualized endometriomas appear exclusively in pregnant women. Mural nodules may show high signal intensity on DWI_800_ due to T2 shine-through effect, but not on higher *b* values, which can help distinguish decidualized endometriomas from ovarian cancers [31].
- *Ovarian metastases* in patients with endometriosis are frequently bilateral. Krukenberg’s tumors are the most common and originate from a primary adenocarcinoma. In order of frequency, the primary tumor site is the stomach, colon-rectum, appendix, pancreaticobiliary tract, contralateral ovary, endometrium (Fig. 10), breast, or lung. Krukenberg’s tumors are classically mixt lesions with solid components displaying low signal on T1- and T2-weighted sequences, diffusion restriction, and enhancement, associated to cystic areas with T2 hyperintensity and no enhancement [32]. Other cancers can also spread to the ovaries, including melanoma or lymphoma. At US, the “lead vessel” sign corresponding to a main peripheral vessel penetrating within the central part of the ovarian mass with a tree-shaped morphology can help to distinguish metastatic from the primary ovarian lesion in which this sign is absent [33].
- *Other ovarian lesions displaying spontaneous high signal intensity T1-weighted*: fat-containing lesions (dermoid cysts), protein-mucinous-containing lesions (mucinous epithelial tumor, abscesses, *struma ovarii*) or hemorrhagic lesions (functional hemorrhagic cyst, ovarian hematoma complicating adnexal torsion) should also be considered as differential diagnoses [34].
- *Desmoid-type fibromatosis* is a rare locally aggressive fibroblastic neoplasm and represents the main differential diagnosis of abdominal wall endometriosis in women with a history of cesarean section. The MRI features depend on its composition but the most commonly observed pattern is iso- or high signal intensity on T2 images, with moderate to intense enhancement and typical low-intensity, non-enhancing linear bands corresponding to dense collagenous stroma [35].

## Treatment

Pain and infertility are the main endometriosis-related symptoms that may lead to consider medical or surgical treatment [36].

In cases of ovarian or extra-ovarian malignancy, the treatment depends on the stage of the disease and often consists of surgery and/or chemotherapy.

Prior to surgical treatment, it is interesting for the surgeon to know whether or not there is a suspicion of associated pelvic endometriosis, as it may mean the presence of pelvic adhesions, increasing the risk of lesions of adjacent organs.

**Fig. 11:**
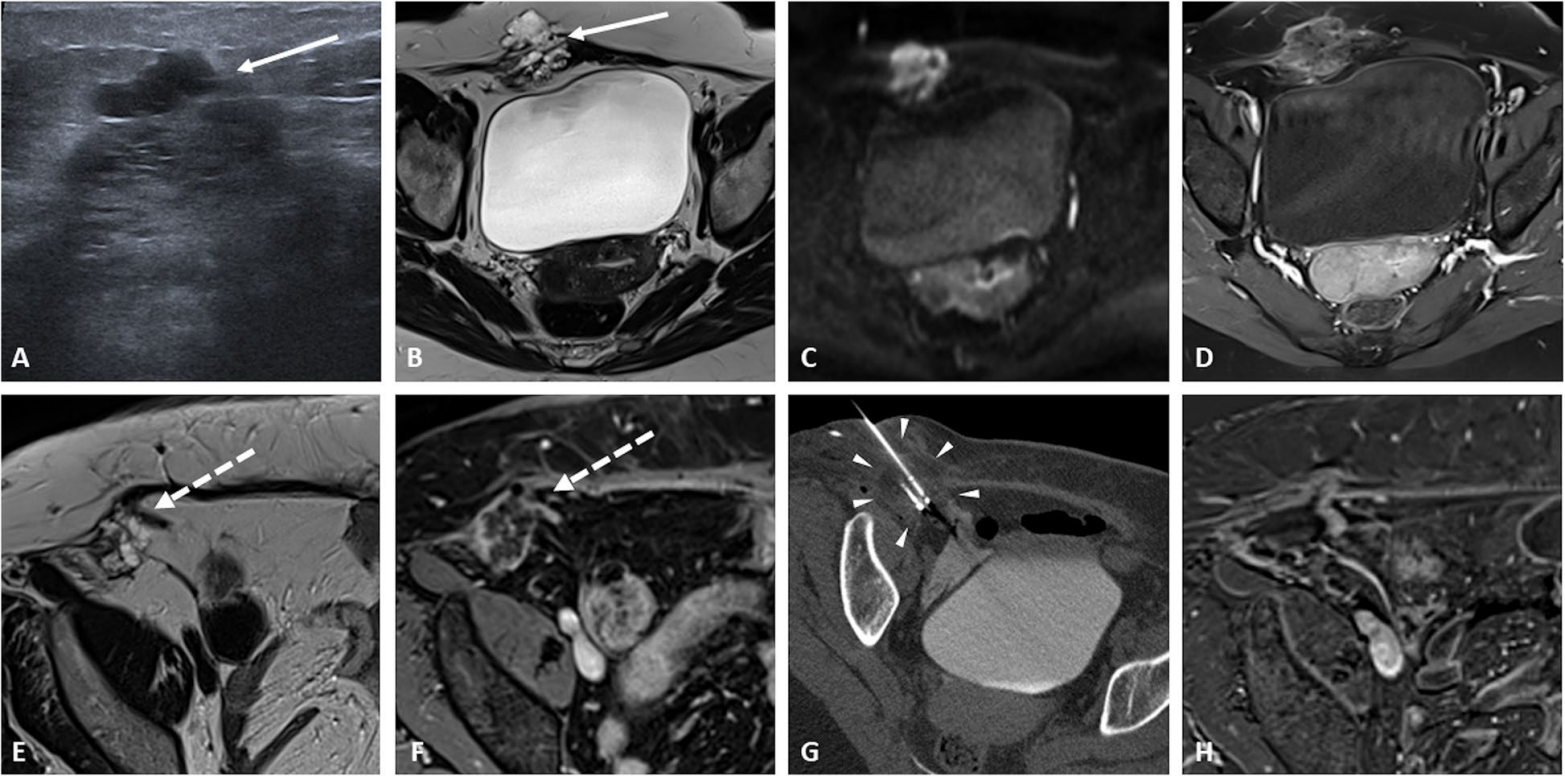
Treatment of an endometriosis-associated clear-cell carcinoma on abdominal wall caesarian scar in a 47-year-old woman. Abdominal wall ultrasound scan (**a**) shows an ill-defined mass containing cystic portions within the rectus abdominis muscle and subcutaneous fat (solid arrows). Further exploration by MRI with axial T2-weighted image (**b**) shows a mixed lesion high signal intensity on diffusion image (**c** high b value) and enhancement of the septae on axial contrast-enhanced fat-suppressed T1-weighted (**d**). The patient benefited from a complete surgical excision and pathology showed a clear cell carcinoma developed on underlying endometrioma. Two years later, local recurrence is shown on the axial T2 image and fat-suppressed enhanced T1 image (dotted arrows in **e** and **f**). Multidisciplinary decision was a conservative percutaneous cryotherapy treatment. The CT scan (**g**) shows the cryoprobe placement and ice formation during treatment (arrowheads). Follow-up MRI scan at 3 months (**h** axial fat-suppressed enhanced subtraction image) shows complete disappearance of the mass

In cases of lesions limited to the abdominal wall, the first results concerning the feasibility and safety of using percutaneous cryotherapy are encouraging but almost exclusively described in non-neoplastic lesions [37] (Fig. 11).

## Follow-up

There are no official guidelines regarding the follow-up of endometriosis. The Nice UK National Guideline Alliance recommends outpatient follow-up for women with confirmed endometriosis (with or without examination or pelvic imaging), particularly women who choose not to have surgery, if they have at least one ovarian endometrioma larger than 3 cm, or deep pelvic endometriosis involving bowel, bladder, or ureter [38].

One study analyzed the time interval between diagnosis of endometrial cyst and associated malignancy: most of the cases evolved to cancer within 120 months [39].

**Fig. 12:**
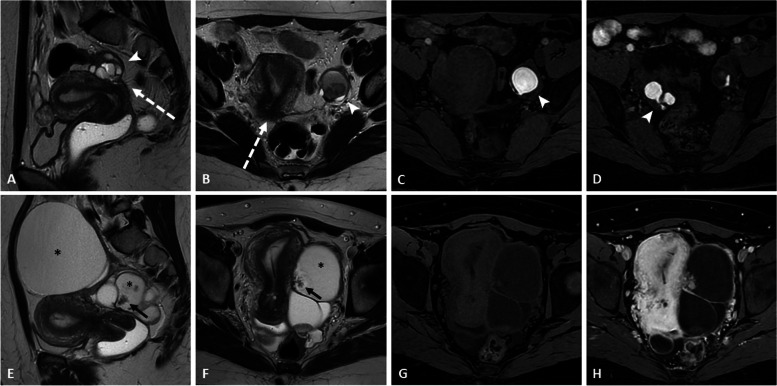
Malignant evolution of an ovarian endometrioma in a 37-year-old woman. Sagittal (**a**) and axial (**b**) T2 images and axial fat-suppressed T1 images (**c**, **d**) show the pelvic endometriosis with torus uterinum thickening (dotted arrows) and typical bilateral endometriomas (arrowheads). A simple clinical surveillance was decided. MRI scan (axial and sagittal T2 images (**e**, **f**) and axial fat-suppressed T1 images before and after injection of gadolinium chelates (**g**, **h**)) performed 6 years later due to a palpable abdominal mass showed a large ovarian mixed lesion with a cystic component (asterisk) and endocystic tumoral nodules (black arrow in **f**). Surgery was performed and found a left ovarian borderline seromucinous carcinoma developed on endometrioma

Imaging follow-up could be proposed for patients with known endometriosis and should be performed whenever a change in symptoms is noted (Fig. 12).

## Conclusion

Concomitant MRI findings of typical endometriosis lesions and suspicious ovarian tumors should raise concern over a potential malignant transformation of benign endometriosis. Subtraction MR imaging plays a key role in identifying solid components within an endometrioma.

### Supplementary Information

**Additional file 1: Fig. S1.** Clear cell carcinoma within an endometrioma. Histological sample with Hps x 20 (A) and Hps x 40 (B) shows a typical endometriotic cyst with a layer of regular cells on endometrial stroma (asterisk), adjacent to a clear cell carcinoma (arrow) showing malignant glands with severe cytonuclear atypia with a pathognomonic “hobnail” appearance. **Fig. S2.** Endometrioid carcinoma within an endometrioma. Histological image (A) showing a typical endometriotic cyst with a layer of regular cells on endometrial stroma (arrowhead in A and B), adjacent to a endometrioid carcinoma (asterisk in A and B) (Hps x 50). Image B: CD10 staining highlighting the endometrial stroma in the endometriotic cyst (Hps x 50). **Fig. S3.** Typical deep-pelvic endometriosis in a 29-year-old woman with infertility. Pelvic ultrasound (A) shows bilateral ovarian endometriomas (asterisk). On axial T1-weighted MRI image (B) endometriomas (asterisks) display high signal intensity, persisting after fat-suppression (C). Coronal (D) and sagittal (E) T2-weighted image show hypointense strands (dotted arrows), joining the ovaries in a "kissing-ovaries" appearance and involvement of the anterior wall of the sigmoid. Corresponding transvaginal Ultrasound scan (F) helps estimate the depth of the digestive involvement. **Fig. S4.** Mixed endometrioid and clear cell carcinoma in a 47-year-old woman with history of surgery for endometriosis. Sagittal (A) and axial T2-weighted images (B) showing a mixed ovarian mass (asterisk) with endocystic peripheral nodules (arrows). There is a loss of the T2-shading intensity usually found in endometriomas. Restricted diffusion regarding the solid nodules is displayed (C: high b value and D: ADC map). Axial fat-suppressed (E) and enhanced fat suppressed (F) T1-images show marked enhancement of the mural nodules, confirmed on dynamic contrast enhancement analysis (type 3 pink curve): suspicion of malignancy. **Fig. S5.** Clear cell carcinoma developed on underlying endometriosis with lymph nodes invasion, and peritoneal and pulmonary metastases in a 27-year-old-woman. Sagittal T2-weighted sequence (A) shows a cystic mass (asterisk) originating from the left ovary with carcinosis of the Douglas pouch and ascites (dotted arrow). Axial T2-weighted image (B) shows peripheral nodules (black arrow) within the ovarian cyst. Axial fat-suppressed T1-weighted image (C) shows a small, left ovarian endometrioma (arrowhead). Subtraction sequence following intravenous injection of gadolinium chelates (D) shows enhancement of the ovarian endometrioma (asterisk) wall (arrow). Diffusion-weighted imaging (E, high b value) reveals multiple metastatic lymph nodes and diffuse peritoneal extension (arrowheads). Complementary chest CT (F) also revealed multiple lung metastases. **Fig. S6.** Differential diagnosis: severe pelvic endometriosis with complex ovarian endometriomas in a 39-year-old woman. Sagittal (A), coronal (B) and axial (C) T2 images show large bilateral ovarian endometriomas (asterisks) with a “kissing-ovaries” appearance (dotted arrow in B). Heterogeneous material is seen within the right endometrioma (white arrows in B and C). On axial fat-suppressed T1 image (D), endometriomas show typical high signal intensity due to hemorrhagic content, whereas solid material is displayed within the right endometrioma (white arrows). Evaluation of intracystic material enhancement is limited on fat-suppressed enhanced T1 image alone (E), whereas subtraction sequence displays well the lack of enhancement of the solid portions within the cyst, corresponding to simple blood clots.

## Data Availability

Not applicable.

## Abbreviations

ADC: Apparent diffusion coefficient
CT: Computed tomography
DCE: Dynamic contrast-enhanced
DWI: Diffusion-weighted imaging
MRI: Magnetic resonance imaging
US: Ultrasound
