# Supplementary material for: Pictorial essay: MRI evaluation of endometriosis-associated neoplasms
Source: Insights Imaging. 2023 Sep 7;14:144. doi: 10.1186/s13244-023-01485-8 (PMC10482819; doi:10.1186/s13244-023-01485-8)

Pictorial essay: MRI evaluation of endometriosis – associated neoplasms

ELECTRONIC SUPPLEMENTARY MATERIAL

Figure S1. Clear cell carcinoma within an endometrioma.

Histological sample with Hps x 20 (A) and Hps x 40 (B) shows a typical endometriotic cyst with a layer of regular cells on endometrial stroma (asterisk), adjacent to a clear cell carcinoma (arrow) showing malignant glands with severe cytonuclear atypia with a pathognomonic “hobnail” appearance.

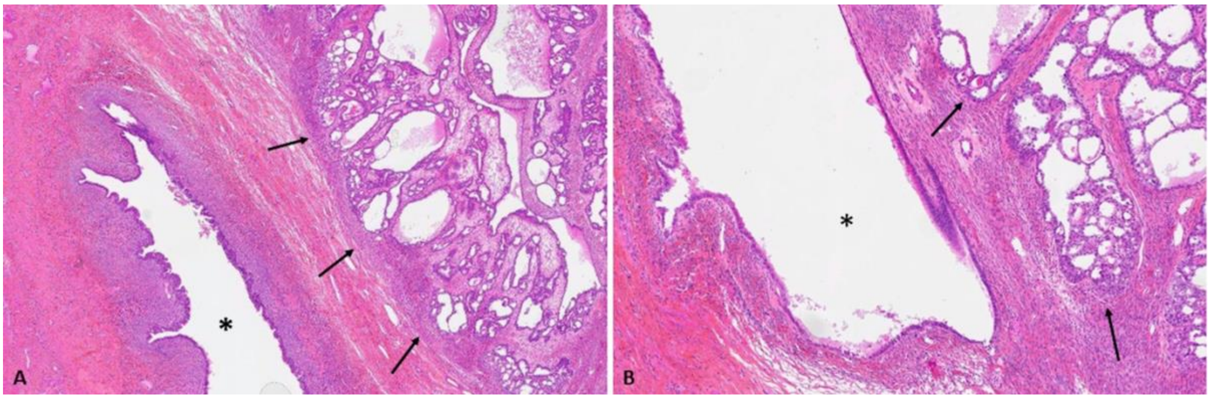

Figure S2. Endometrioid carcinoma within an endometrioma.

Histological image (A) showing a typical endometriotic cyst with a layer of regular cells on endometrial stroma (arrowhead in A and B), adjacent to a endometrioid carcinoma (asterisk in A and B) (Hps x 50). Image B: CD10 staining highlighting the endometrial stroma in the endometriotic cyst (Hps x 50).

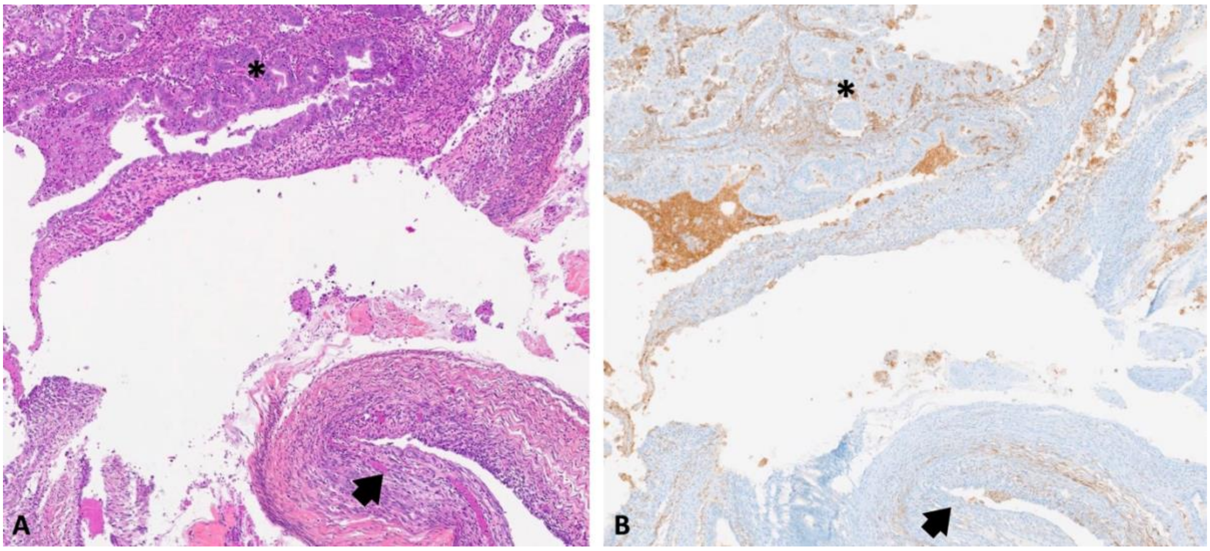

Figure S3. Typical deep-pelvic endometriosis in a 29-year-old woman with infertility.

Pelvic ultrasound (A) shows bilateral ovarian endometriomas (asterisk). On axial T1-weighted MRI image (B) endometriomas (asterisks) display high signal intensity, persisting after fat-suppression (C). Coronal (D) and sagittal (E) T2-weighted image show hypointense strands (dotted arrows), joining the ovaries in a "kissing-ovaries" appearance and involvement of the anterior wall of the sigmoid. Corresponding transvaginal Ultrasound scan (F) helps estimate the depth of the digestive involvement.

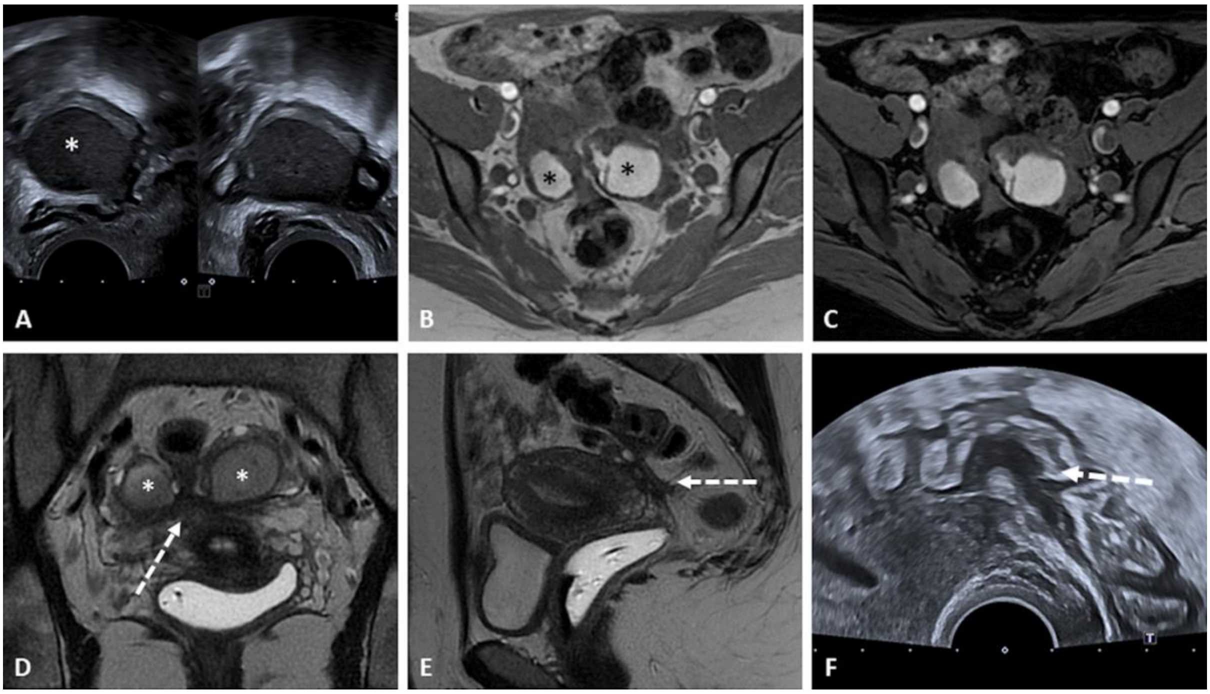

Figure S4. Mixed endometrioid and clear cell carcinoma in a 47-year-old woman with history of surgery for endometriosis.

Sagittal (A) and axial T2-weighted images (B) showing a mixed ovarian mass (asterisk) with endocystic peripheral nodules (arrows). There is a loss of the T2-shading intensity usually found in endometriomas. Restricted diffusion regarding the solid nodules is displayed (C: high *b* value and D: ADC map). Axial fat-suppressed (E) and enhanced fat suppressed (F) T1-images show marked enhancement of the mural nodules, confirmed on dynamic contrast enhancement analysis (type 3 pink curve): suspicion of malignancy.

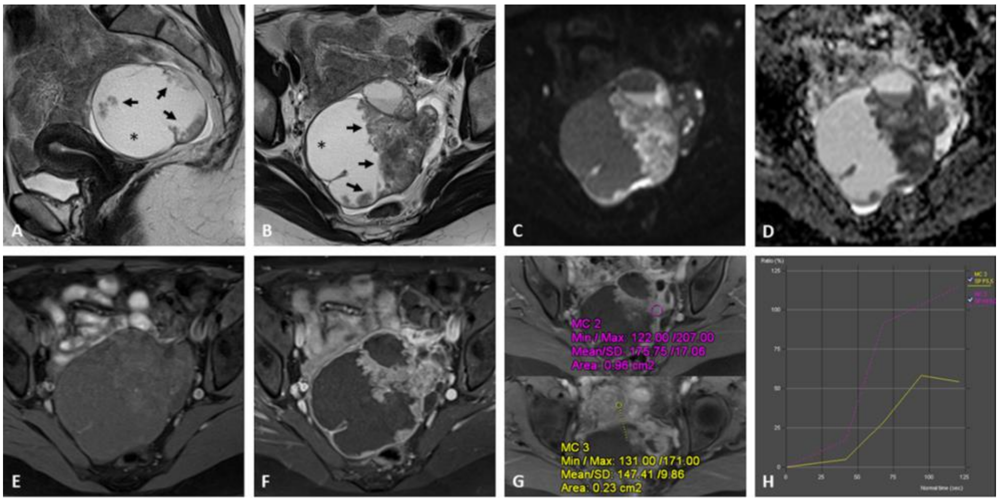

Figure S5. Clear cell carcinoma developed on underlying endometriosis with lymph nodes invasion, and peritoneal and pulmonary metastases in a 27-year-old-woman. Sagittal T2-weighted sequence (A) shows a cystic mass (asterisk) originating from the left ovary with carcinosis of the Douglas pouch and ascites (dotted arrow). Axial T2-weighted image (B) shows peripheral nodules (black arrow) within the ovarian cyst. Axial fat-suppressed T1-weighted image (C) shows a small, left ovarian endometrioma (arrowhead). Subtraction sequence following intravenous injection of gadolinium chelates (D) shows enhancement of the ovarian endometrioma (asterisk) wall (arrow). Diffusion-weighted imaging (E, high *b* value) reveals multiple metastatic lymph nodes and diffuse peritoneal extension (arrowheads). Complementary chest CT (F) also revealed multiple lung metastases.

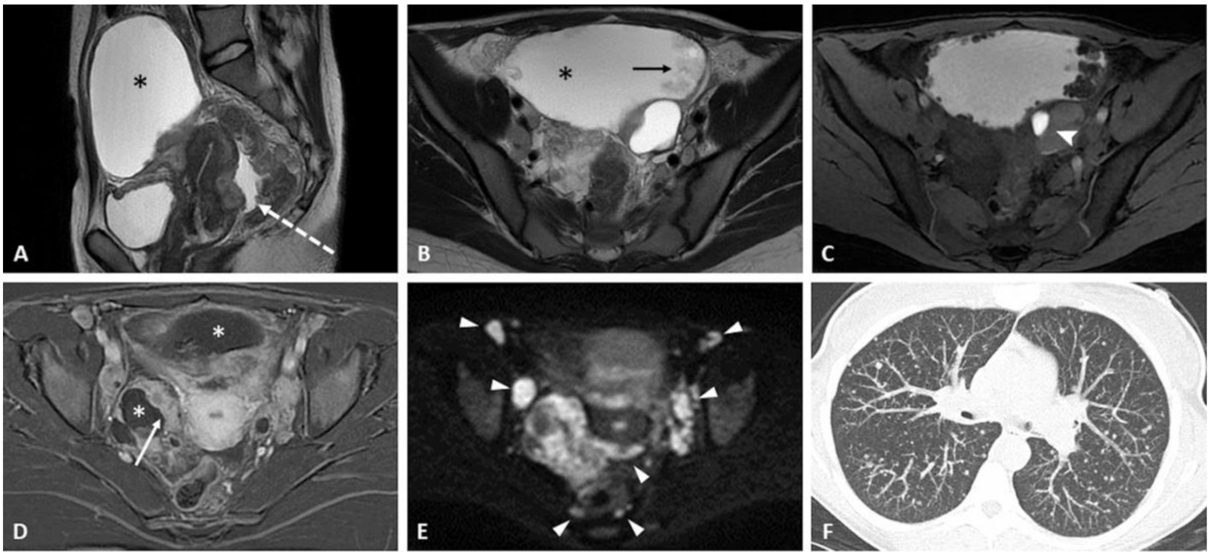

Figure S6. Differential diagnosis: severe pelvic endometriosis with complex ovarian endometriomas in a 39-year-old woman.

Sagittal (A), coronal (B) and axial (C) T2 images show large bilateral ovarian endometriomas (asterisks) with a “kissing-ovaries” appearance (dotted arrow in B). Heterogeneous material is seen within the right endometrioma (white arrows in B and C). On axial fat-suppressed T1 image (D), endometriomas show typical high signal intensity due to hemorrhagic content, whereas solid material is displayed within the right endometrioma (white arrows). Evaluation of intracystic material enhancement is limited on fat-suppressed enhanced T1 image alone (E), whereas subtraction sequence displays well the lack of enhancement of the solid portions within the cyst, corresponding to simple blood clots.

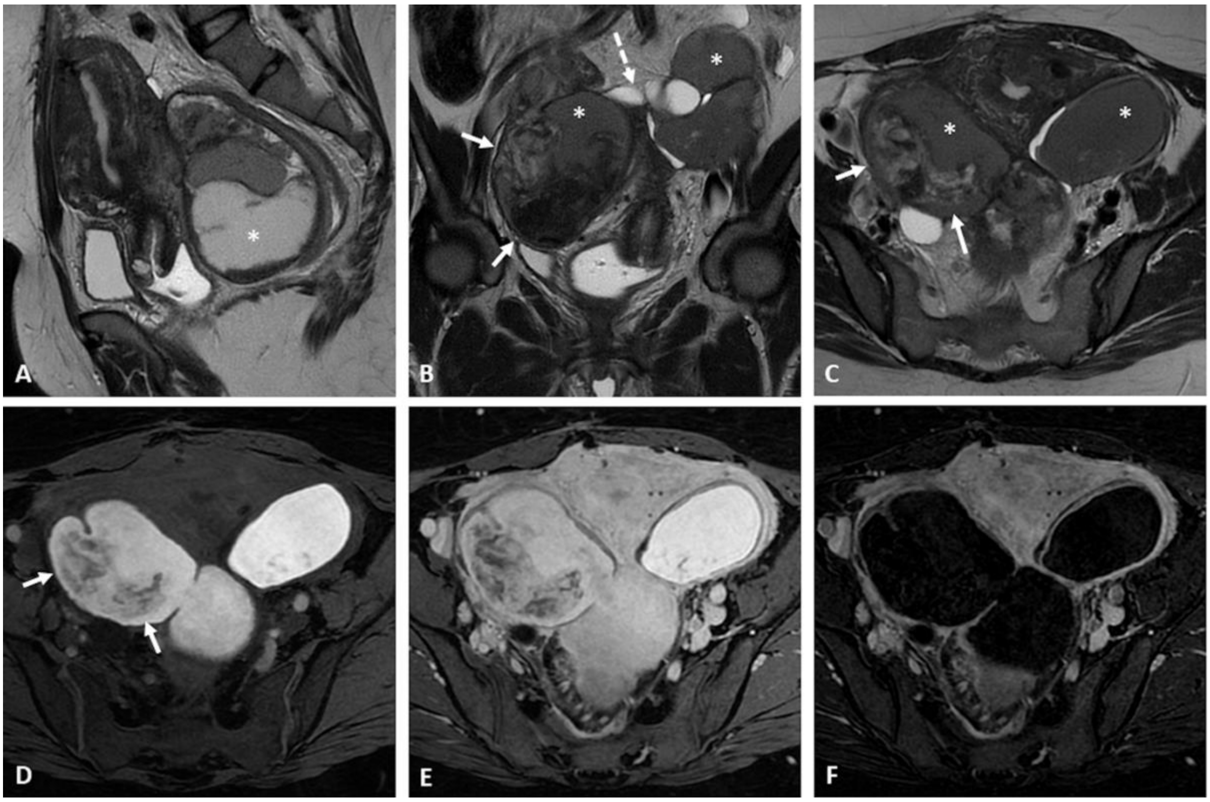

Supplement: Supplementary file 1 — Additional file 1: Fig. S1. Clear cell carcinoma within an endometrioma. Histological sample with Hps x 20 (A) and Hps x 40 (B) shows a typical endometriotic cyst with a layer of regular cells on endometrial stroma (asterisk), adjacent to a clear cell carcinoma (arrow) showing malignant glands with severe cytonuclear atypia with a pathognomonic “hobnail” appearance. Fig. S2. Endometrioid carcinoma within an endometrioma. Histological image (A) showing a typical endometriotic cyst with a layer of regular cells on endometrial stroma (arrowhead in A and B), adjacent to a endometrioid carcinoma (asterisk in A and B) (Hps x 50). Image B: CD10 staining highlighting the endometrial stroma in the endometriotic cyst (Hps x 50). Fig. S3. Typical deep-pelvic endometriosis in a 29-year-old woman with infertility. Pelvic ultrasound (A) shows bilateral ovarian endometriomas (asterisk). On axial T1-weighted MRI image (B) endometriomas (asterisks) display high signal intensity, persisting after fat-suppression (C). Coronal (D) and sagittal (E) T2-weighted image show hypointense strands (dotted arrows), joining the ovaries in a "kissing-ovaries" appearance and involvement of the anterior wall of the sigmoid. Corresponding transvaginal Ultrasound scan (F) helps estimate the depth of the digestive involvement. Fig. S4. Mixed endometrioid and clear cell carcinoma in a 47-year-old woman with history of surgery for endometriosis. Sagittal (A) and axial T2-weighted images (B) showing a mixed ovarian mass (asterisk) with endocystic peripheral nodules (arrows). There is a loss of the T2-shading intensity usually found in endometriomas. Restricted diffusion regarding the solid nodules is displayed (C: high b value and D: ADC map). Axial fat-suppressed (E) and enhanced fat suppressed (F) T1-images show marked enhancement of the mural nodules, confirmed on dynamic contrast enhancement analysis (type 3 pink curve): suspicion of malignancy. Fig. S5. Clear cell carcinoma developed on underlying e [file 13244_2023_1485_MOESM1_ESM.pdf]
